# Effect of Refractance Window^™^ and oven drying on physicochemical and sensory properties of peach (*Prunus persica* L.) surplus

**DOI:** 10.3389/fnut.2024.1307423

**Published:** 2024-04-23

**Authors:** Esteban Largo-Avila, Fabián Rico-Rodríguez, Jeanine Kathleen Peñaloza-Figueroa, Alexis López-Padilla

**Affiliations:** ^1^Caicedonia Regional Campus, Universidad del Valle, Cali, Colombia; ^2^Food Engineering Department, Universidad de Cartagena, Cartagena de Indias, Colombia; ^3^Processes and Energy Department, Universidad Nacional de Colombia, Medellín, Colombia

**Keywords:** Refractance Window drying, food surplus, fruit valorization, sensory analysis, food security

## Abstract

Currently, approximately 34% of fruit is lost and wasted in emerging economies due to inefficient post-harvest processes, technological shortcomings, lesser valorization of surpluses, and byproducts. Peach (*Prunus persica* L.) is a fruit with a good yearly growth rate but higher postharvest losses in Colombia. One way to take advantage of this type of product is through the application of drying processes that increase its shelf life and its inclusion in the food chain. Refractance Window Drying (RWD) is a fourth generation drying technique implemented by the food industry in the last few decades and has been applied to several dehydrated food products. This study compared the effects of different drying methods on the physical and sensory properties of peaches surplus. Treatments consisted of (i) peaches were sliced (1, 2, and 3 mm thickness) and dried using RWD at 86°C, (ii) peach pulp mixed with maltodextrin (MD) (0.12–0.33 kg of MD/kg of sample) and RWD (RWD-MD), and (iii) conventional oven drying (OD) at 60°C (3 mm thick) dried for 24 h. The study found that the drying method significantly (*p* < 0.05) affected the texture, color, and general taste of peaches. The results showed that processing treatments combined with different drying conditions affected the physical properties of the peach. RWD in slices reduced water content to 0.05 kg H_2_O/kg in 40 min, showing fewer effects on color attributes. A surface response analysis on RWD showed good correlations for water activity (*R*^2^ = 0.8652–0.9894) and moisture content (*R*^2^ = 0.7048–0.9826). A higher diffusion coefficient (1.63 × 10^−6^ m^2^ s^−1^) was observed for RWD in slices with 3 × 10^−3^ m of thickness; however, for RWD-MD, differences in diffusion coefficients were present for the lowest MD addition (0.12 kg/kg), vitamin C was not detected on the dried slices, and higher concentration of β-carotene (175.88 μg/100 g) was found on the thinner slices. Principal component analysis showed that RWD in the slices was the most suitable drying process, followed by OD. Sensory analysis showed good acceptability for RWD slices after 30 days of storage.

## 1 Introduction

Approximately 40% of the total food production is lost due to waste in the food supply chain, which worsens the global food crisis and has a negative impact on the environment. Despite the severe food shortage, the majority of this waste occurs in emerging economies due to a lack of suitable and economical preservation methods (1).

Peach (*Prunus persica* L.) is a fruit that is widely available worldwide and is known for its delicacy and perishable nature. In 2022, China was the largest producer of peaches (over 19 million tons), representing 64% of the world’s production of peaches and nectarines (2). *Prunus persica* is one of the most important fruits in tropical countries with high elevations. In Colombia, peach fruits are grown in areas with average temperatures between 13°C and 19°C, located between 1,800 and 2,800 meters above the sea level (3).

According to FAOSTAT, in 2020, peach production in Colombia was estimated at 31.386 tons, with a yearly growth rate of 4.5%, showing increasing interest in industries and local consumers. These market behaviors have a disadvantage in emerging economies such as Colombia, where there are high post-harvest losses and expensive waste management treatments (2) due to insufficient post-harvest processes, gaps in technology, and low value-added products. However, inadequate post-harvest treatments or processes are responsible for approximately 34% of the losses in peach and nectarine production, followed by low technological alternatives for the valorization of residues, by-products, or production surpluses (4–6). In this context, the drying process is an economic alternative to taking advantage of the recent high production of peaches in Colombia, transforming surplus or rejected fruits into dried products, increasing their shelf life, and creating strategies that can be incorporated into a productive process strategy by adding value to *P. persica*.

Drying agricultural products is an economical and effective preservation method to reduce water content and increase shelf life (7). Within the literature concerning drying processes, numerous studies are dedicated to the utilization of oven or hot-air drying for various applications, oven drying (OD) is one of the most used in the food industry because of its low cost (8), but the long processing time and the use of high air temperatures produce alterations or adverse effects on their sensory qualities (9, 10).

To preserve the physical, chemical, and sensory properties of peaches, various technologies and drying methods have been employed. Chatzilia et al. (11) and Roknul Azam et al. (12) combined hot-air convection and microwave drying. Pieniazek and Messina (13) utilized freeze-drying, while Lyu et al. (14) and Zhang et al. (15) initiated the process with osmotic dehydration, followed by infrared pre-drying, and concluded with explosion puffing. In the study by Doymaz and Bilici (16), peaches were pre-treated with citric acid before undergoing convection drying with hot air. Additionally, Zhu and Shen (17) employed hot-air convection drying, and Wang and Sheng (18) used a combination of far-infrared and microwave drying techniques in peaches.

Currently, there is a trend toward using improved drying methods that are faster, more uniform, hygienic, and require less energy than conventional methods (11, 12). However, the selection of a suitable drying technology is a challenging task because fruits and vegetables are susceptible to heating; conductive hydro-drying, such as Refractance Window Drying (RWD), is a novel fourth generation of drying methods with a relatively short processing time, low energy, low cost, scalability, and improved product quality (19–22).

As outlined by researchers, the RWD method involves the use of hot water, reaching temperatures of up to 98°C under atmospheric pressure conditions. This process facilitates the transfer of thermal energy to the product, typically resulting in a viscous suspension, pasty food, puree, or sliced form. The treated product is then arranged on a polyethylene terephthalate film or a similarly infrared radiation-transparent material, which is placed in direct contact with the hot water. Water transfers heat energy through conduction and convection heat-transfer mechanisms. Water is usually recirculated to improve the thermal efficiency of the process. The thermal energy of the water passes through a plastic film by conduction and radiation, the moisture in the product is removed by the air flowing over the food layer, and the product reaches temperatures below 70°C (23–25).

Based on several years of research, the RWD process was developed by MCD Technologies, Inc. (Tacoma, Washington) in novel water evaporating techniques with the applications for the drying of egg white, avocado (*Persea americana*), mango (*Mangifera indica* L.), Kiwifruits (*Actinidia deliciosa*), microalgae (*Spirulina plantensis*), Jackfruit (*Artocarpus heterophyllus* L.), meat, bone broth powder, herbal extracts and supplements, and dried fruits and vegetables (8, 25–33). In addition to the advantages of RWD in producing high-quality products, as stated above, RWD can also reduce energy use and improve the stability of probiotics in powder form, such as *Lactobacillus plantarum* (NCIM 2083), creating a new approach for their commercialization (34).

During drying of fruits, some changes could appear, depending on the process and affecting the physical and sensory properties of the raw material and the global quality of the final product. The moisture content and the water activity have been studied in several drying processes because these characteristics are determinant on the quality of a product and its microbial safety (32, 35, 36).

Color plays an important role in the sensory acceptance of dried products because it involves good drying processes and has a strong influence on consumer purchase decisions and product value (37). Visual recognition of a material and its subsequent assessment are part of a global appearance described by the color, and this is the first quality judgment made by a customer (38); for this reason, color is the most important appearance attribute (39).

The main goal of this study was to evaluate the effects of Refractance Window^™^ drying and conventional oven drying on the moisture content, water activity, mass transfer, ascorbic acid retention, color properties, and sensory acceptability of peach fruit (*Prunus persica* L.) surpluses harvested in Colombia.

## 2 Materials and methods

### 2.1 Sample processing and physicochemical analysis

Fresh and mature peach (*Prunus persica* L.) surpluses or rejected were purchased from a local market according to the Colombian Technical Standard for fresh fruits and vegetables NTC 1291:1977 (40), following the quality standards for Colombian peaches (41). The fruits were washed and disinfected with the organic sanitizer Citrosan^®^ and then separated into three batches.

- Batch 1: Fruits were sliced into 1, 2, and 3 × 10^−3^ m of thickness with a mean diameter of 0.05 m and was vacuum packed in foil zip sealed pouches (BOPP/polyamide/LDPE) until drying.
- Batch 2: The fruits were ground in a cutting machine with 1.5 × 10^−2^ m^3^ of capacity (Cruells, Girona, Spain) at a chopper speed of 1,300 rpm; the pulp obtained was mixed with maltodextrin (MD) from 0.12 to 0.33 kg/kg of peach pulp (based on preliminary experiments), vacuum packed, and stored until drying.
- Batch 3: The fruit was sliced into 1, 2, and 3 × 10^−3^ m thickness and then disposed in aluminum trays of 0.40 m × 0.60 m and dried in an conventional oven drying.

#### 2.1.1 Physicochemical analysis

Titratable acidity was measured using 10 g of sample properly diluted in 90 mL of distilled water and tritrated with NaOH (0.1 N), and the results were expressed as grams of malic acid per 100 mL of sample. The pH was measured using a Handylab pH/LF 12 pH meter (SI Analytics GmbH, Mainz, Germany). Moisture content was measured using 1.0 × 10^−3^ kg of sample in a halogen moisture analyzer (OHAUS MB35, OHAUS Corporation, New Jersey, United States), with halogen light at a constant temperature of 105°C. The total soluble solids were measured using a digital hand-held “Pocket” refractometer ATAGO PAL-3 (Atago Co., Ltd., Tokyo, Japan). Data are given in °Brix. Sugars were measured by the UV–VIS Spectrophotometer method using the 3,5-dinitrosalicylic acid (DNS) method (42, 43). Total dietary fiber of fresh pulp was measured with a gravimetric method AOAC 993.21 (44). The energy of the fresh pulp was measured using the calorimetric method ISO 9831 and expressed in calories (45).

### 2.2 Drying methods

#### 2.2.1 Refractance Window^TM^ drying

The RWD dryer consisted of a 5.50 × 10^−3^ m^3^ stainless steel thermostatic bath filled with tap water. The water surface was covered with Mylar^™^ (an infrared transparent polyethylene terephthalate plastic film with 2.60 × 10^−4^ m of thickness with an area of 0.05 m^2^), on which the samples were placed. The temperature of the bath was set at 86 ± 0.5°C, based on the atmospheric pressure in the city of Medellin, Colombia (640 mm of Hg at 1530 m.a.s.l), following other results reported in the literature (23, 35, 46). A factorial experimental design was carried out to evaluate the effect RWD of samples with three levels of thickness 1 × 10^−3^ m, 2 × 10^−3^ m and 3 × 10^−3^ m and a second factorial design based on central composite designs (CCD) was built to evaluate the effect of maltodextrin (MD) addition levels from 0.15 to 0.30 kg/kg of peach pulp based on preliminary experiments to accelerate the drying process and setting a layer of ~2 × 10^−3^ m of thickness for all samples and including 2 axial points (−*α* = 0.12 kg/kg and +*α* = 0.33 kg/kg). MD has been used as a carrier material to decrease the drying rate and increase the glass transition temperature in different products such as mango (47) and avocado pulp (48).

#### 2.2.2 Oven drying

Peach fruit slices were placed on a conventional oven dryer FD53-UL from Binder GmbH (Tuttlingen, Germany) with 3 × 10^−3^ m of thickness, and the drying temperature was fixed at 60°C for 24 h at 1.5 m s^−1^ of air speed until a constant weight was attained, which was chosen based on previous experiments (Table 1).

**Table 1:** Experimental design for peach (*Prunus persica* L.) drying process by Refractance Window^™^ at 86°C and oven drying at 60°C.

Drying process	Factor	Levels		
		−1	0	+1
Refractance Window drying, 92°C	Thickness (m × 10^−3^)	1	2	3
	Drying time (min)	10	35	60
	Maltodextrine addition (kg/kg)	0.15	0.23	0.30
Oven drying 60°C	Thickness (m × 10^−3^)	—	3	—

Response variables for all experiments were water content (*H*, %), water activity (*a*_w_), and color coordinates (L^*^, *a*, ^*^, and b^*^) and its computed *h* and C^*^.

#### 2.2.3 Moisture content and water activity

The moisture content of the dried samples was measured using a moisture analyzer OHAUS MB35 from the OHAUS Corporation (Newark, NJ, United States) with a constant temperature of 100°C. The water activity ( $a_{w}$ ) of the dried samples was measured using a water activity meter Aqualab 3TE from Decagon Devices, Inc. (Pullman, WA, United States), which applies the dew point method, where water is condensed on the clear and cold surface of a mirror and is detected by an infrared sensor.

#### 2.2.4 Mathematical modeling

The mathematical model of the drying kinetics was developed by Fick’s second law using the humidity ratio (HR) (see Eq. 1):


(1) $$HR=\frac{W_{t}-W_{e}}{W_{0}-W_{e}}$$

where *W*_t_*, W*_0_, and *W*_e_ are time *t* in (s), initial, and equilibrium humidity content (dry basis, db), respectively. As described by other authors (20, 49), when the RW equipment cannot control the relative moisture of the air in the experimental area, *W*_e_ becomes negligible with respect to *W*_t_ and *W*_0_, and Eq. 1 can be simplified to obtain Eq. 2:


(2) $$HR=\frac{W_{t}}{W_{0}}$$

The effective diffusivity of the humidity was calculated by Fick’s diffusion law using the equation proposed by Crank in 1975 (50), Eq. 3:


(3) $$HR=\frac{W_{t}-W_{e}}{W_{0}-W_{e}}=\frac{8}{\pi^{2}}\sum_{n=0}^{\infty }\frac{1}{{\left(2k+1\right)}^{2}}\exp\left(-\frac{{\left(2k+1\right)}^{2}\pi^{2}D_{\mathrm{eff}}t}{4L_{0}^{2}}\right)$$

where *D*_eff_ represents the effective humidity diffusion in m^2^ s^−1^, *L*_0_ is the thickness of the slice in (m), and *k* is a positive integer. Eq. 3 can be simplified to a linear equation, as shown in Eq. 4.


(4) $$\mathrm{lnln}\;\left(HR\right)=\ln\frac{8}{\pi^{2}}-\frac{D_{\mathrm{eff}}\pi^{2}t}{4L_{0}^{2}}$$

The plot of Eq. 4 results in a negative slope *S*, which correlates with the diffusion coefficient, according to Eq. 5:


(5) $$S=\frac{D_{\mathrm{eff}}\pi^{2}}{4L_{0}^{2}}$$

#### 2.2.5 Color

The instrumental color of each dried sample was measured at different points on the surface by a CIELab system with a colorimeter X-Rite SP64 from X-Rite, Inc. (Michigan, United States) using a D_65_ illuminant, a 10° observer angle with an aperture size of 6.40 × 10^−2^ m. Color was described as coordinates: lightness (L^*^), redness (a^*^, red-green), and yellowness (b^*^, yellow-blue); the parameters derived Chrome (C^*^) using the Eq. 6 and Hue angle (h^*^) using the Eq. 7, and the numerical total color difference or total deviation (Δ*E*) was calculated using the Eq. 8.


(6) $$C^{*}={\left[{\left(a^{*}\right)}^{2}+{\left(b^{*}\right)}^{2}\right]}^{\frac{1}{2}}$$

(7) $$h=\tan^{-1}\left(\frac{b^{*}}{a^{*}}\right)$$

(8) $$\Delta E=\sqrt{{\left(L_{0}^{*}-L^{*}\right)}^{2}+{\left(a_{0}^{*}-a^{*}\right)}^{2}+{\left(b_{0}^{*}-b^{*}\right)}^{2}}$$

In the Eq. 8, the subscript “0” means the color of the pulp peach. When the average Δ*E* difference between two samples is equal to or less than 2.56, the samples are considered not discriminable from each other by a judge or sensory panel (51).

#### 2.2.6 Vitamin C and carotenoid analysis

Vitamin C content was determined by HPLC column (Shimatzu Prominence 20A): Luna^®^ 5 μm C18(2) 100A, 250 × 4.6 mm, mobile phase: KH_2_PO_4_ 0.02 M (pH: 3.06), flow rate: 1 mL/min, pressure: 1172 psi, retention times: 4.317–4.456 min, injection volume: 5 μL, and wavelength: 244 nm. An analytical standard, l-ascorbic acid (Sigma-Aldrich 47863), was used.

The quantification of total carotenoids was performed as previously described by Biswas et al. (52). In total, 100 mg of sample was placed in a test tube, 4 mL of cold acetone was added, shaken vigorously for 120 s, and allowed to stand for 15 min at 4°C. The mixture was then centrifuged for 10 min, and the supernatant was transferred to another test tube. This procedure was repeated until the sample was exhausted. Finally, acetone extracts were combined and filtered.

The absorbance of the solution was measured at 449 nm by acetone as a blank using a UV/VIS Multiskan Spectrum spectrophotometer LAMBDA 35 (PerkinElmer, Inc., United States). The results were expressed as micrograms of β-carotene per 100 g of sample, using a calibration curve with a β-carotene standard (Sigma-Aldrich, >98%).

#### 2.2.7 Sensory analysis

A sensory analysis was conducted following the score scale recommended by Colombian Technical Standard Normativity NTC 3932 and NTC 5328 (53, 54) and applied to the best drying results obtained in this study. The main goal of this test was to evaluate the characteristics of color, odor/aroma, objectionable odor/aroma, hardness, chewiness, crunchiness, and overall quality with respect to time. Sensory descriptors were previously selected for the product evaluation and scored on a seven-point intensity scale, where 0 = absent, 1 and 2 = mild, 3 = medium-low, 4 = medium, 5 = medium-high, and 6 and 7 = intense. A trained panel formed by five judges analyzed and scored the sensory attributes and overall acceptability of dried peach depending on storage time (1, 15, 31, and 45 days). Samples were packed by 0.10 kg each in plastic bags (BOPP-polyamide-polyethylene) with 120 μm of thickness and stored at 25°C for 45 days.

#### 2.2.8 Data analysis

Data were analyzed by statistical software R-project version 4.2.2 (The R Foundation, https://www.r-project.org/) and Microsoft Excel^®^. OD data were analyzed by one-way ANOVA using a 95% confidence level. Each point was carried out by triplicate. In the case of RWD, a factorial Central Composite Design (CCD) was used with three central points and two levels (2^2^). The levels (−1 and 1) were 60 and 160 min for time and 0.15 and 0.30 kg/kg of peach pulp for the addition of MD. There were two axial points (−*α* = 0.12 kg/kg and +*α* = 0.33 kg/kg), as shown in Table 1. The dependent variables were color (L^*^, a^*^, b^*^, C^*^, h^*^, and Δ*E*), moisture content (*H*, %), and water activity ( $a_{w}$ ); the analysis was made by surface response methodology with a significance level of 0.05. Finally, all data were analyzed by principal component analysis (PCA) as an exploration tool to determine the variability contributions of each parameter on global color appearance. The sensory analysis data were processed using an Excel spreadsheet.

## 3 Results and discussion

### 3.1 Physical and chemical characteristics of peach

Table 2 shows the physical and chemical characterization of the fresh peach, where the values of pH and acidity were similar to those reported by Abidi et al. (55), Grigelmo et al. (56), and Mokrani et al. (57) for peach fruits cultivated in Spain and Algeria, which ranged from 0.154 to 0.5 g of malic acid/100 g of sample. The energy value found in this study was approximately 4 kcal/kg which is slightly a little higher than the values reported by Grigelmo-Miguel et al. (56) (3.5–3.7 kcal/g); differences could be associated with the high sugar content explained by the °Brix values and total sugar content of 13.84 °Brix and 60%, respectively. These results were higher than the values reported for early ripening of peach and varieties of nectarines such as “Filina,” “Ufo-4,” and “Gergana” from Eastern Europe (58) and peaches harvested in the north of China (59, 60). On the other hand, the values found in this study were less than those reported by the study mentioned in the reference (55) for peaches from northern Spain, showing that there are many factors affecting their physicochemical parameters, such as the stage of maturity at picking, storage conditions, origin, or the physiological effects caused by seasonal conditions in the northern hemisphere, which are absent in Colombia. Regarding the vitamin C content, peach fruits are not common sources of vitamin C. In this study, the results showed that non-treated peach fruits (7.2 mg/100 g) were generally in the same range as European cultivars (61). Water content values, on wet basis, in fresh fruit were similar to data published by Abidi et al. (55), Zhou et al. (62), and Dagdelen et al. (63).

**Table 2:** Physicochemical analysis of Colombian peach (*Prunus persica* L.) surpluses.

Physicochemical parameter	Value
Soluble solids (° Brix)	13.84 ± 0.13
pH	3.76 ± 0.06
Acidity (g malic acid/100 g sample)	0.06 ± 0.02
Water content (wet basis, %)	83.80 ± 1.32
Total sugar (%)	59.91 ± 3.7
Total dietary fiber (%)	15.2 ± 0.7
Calories (kcal/kg)	4.106 ± 0.08
Carotenoids (μg β-carotene/100 g)	272.64 ± 2.23
Ascorbic acid (mg/100 g)	7.40 ± 0.02
L^*^	74.22 ± 4.11
a^*^	19.09 ± 2.98
b^*^	58.78 ± 6.87

### 3.2 Drying methods

#### 3.2.1 Refractance Window^™^ drying of peach slices

Tables 3 and 4 show the CCD experimental design results for the RWD slices and RWD with Maltodextrin addition (RWD-MD). For RWD slices, where the moisture content and water activity reduction from the fresh peach attained values between 7% and 26% (dry basis) and 0.295 – 0.755, respectively, indicating a fast and good effect of the RWD method on peach slices (see Table 3)

**Table 3:** Experimental design CCD for RWD of peach fruit in slices and results for water content dry basis (*H*, %) and water activity (*a*_w_).

Order	Thickness (X_1_)	Time (X_2_)	Thickness (m × 10^−3^)	Time (min)	*H* (%)^*^	*a*_w_
1	−1	0	1	35	9.89	0.327
2	+1	+1	3	60	9.89	0.368
3	−1	−1	1	10	20.90	0.669
4	+1	0	3	35	10.43	0.295
5	0	0	2	35	13.88	0.497
6	0	+1	2	60	7.06	0.483
7	0	0	2	35	7.70	0.346
8	+1	−1	3	10	9.10	0.312
9	0	−1	2	10	26.08	0.755
10	−1	+1	1	60	9.57	0.375
11	0	0	2	35	10.79	0.425

^*^Dry basis.

**Table 4:** Analysis of variance and regression models for moisture content and water activity of RWD peach fruit in dependence of thickness, MD addition, and the drying time.

ANOVA					
Sample treatment	Factor	Moisture content (%)		Water activity (*a*_w_)	
		*F*-ratio	*p*-value	*F*-ratio	*p*-value
Slices	*ε*: Thickness (m)	0.97	0.3710	4.30	0.0928
	*t_ε_*: Time (min)	7.05	0.0452^*^	7.13	0.0443^*^
	*ε* × *t_ε_*	1.78	0.2400	5.04	0.0748
MD addition	MD: MD (kg/kg)	30.83	0.0026^*^	164.06	0.0001^*^
	*t*_MD_: Time (min)	199.46	0.0000^*^	276.37	0.0000^*^
	MD × t_MD_	4.82	0.0795	0.85	0.3982

Regression models					
RWD slices			RWD + MD		
Independent variable	Moisture content (%)	Water activity (*a*_w_)	Independent variable	Moisture content (%)	Water activity (*a*_w_)
Constant	28.16	0.63	Constant	15.73	0.76
*ε* (m)	3.98	0.42	MD	37.29	0.46
*t_ε_* (min)	−0.88	−0.03	*t*_MD_	−0.20	−5.82 × 0^−3^
*ε* × *t_ε_*	0.12	0.0035	MD × t_MD_	−0.21	−2.56 × 10^−3^
*ε*^2^	−2.51	−0.15	MD^2^	10.80	2.41
$t_{\varepsilon }^{2}$	0.01	2.50 × 10^−4^	$t_{MD}^{2}$	7.79 × 10^−4^	1.80 × 10^−6^
*R*^2^	0.71	0.86	*R*^2^	0.98	0.99

^*^Factors with significance effect (*p* < 0.05). MD, maltodextrine.

An analysis of both designs was performed using the response surface methodology and the results can be seen in Table 4. For RWD slices, the time has a strong significant effect (*p* < 0.05) on moisture content and water activity, showing similarities with other authors (21, 26, 35), indicating that RWD has a great advantage in fruit drying with short time of processing. Otherwise, thickness has no effect on moisture content and water activity (*p* = 0.3710) and could be associated to the gap between the thickness levels used in this work, on the other hand, these results are suitable for small and medium industries where the equipment for slicing or processing is not enough advanced or automated and the operations must be done manually.

Good correlation coefficient *R*^2^ of 0.705 and 0.865 explained the goodness of fit of the experimental data in the response surface models of moisture content and water activity, respectively, for RWD slices, showing that between 70 and 86% of the total variation was accounted for the two response surface models with data on goodness of fit and explaining adequately the phenomenon involved.

**Figure 1:**
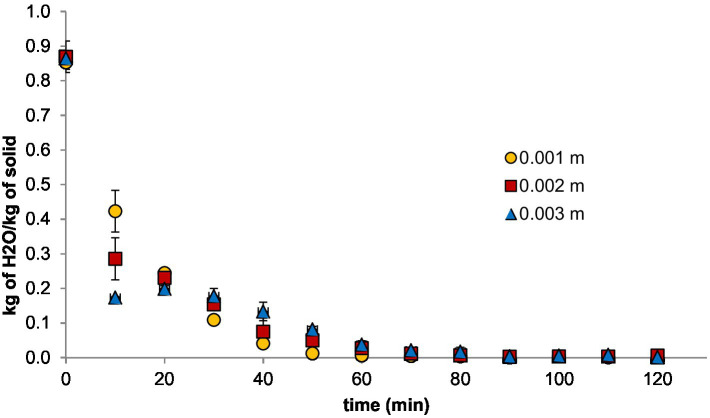
Kinetics of moisture content for RWD, where a = RWD slices with 1 × 10^−3^ m, 2 × 10^−3^ m, and 3 × 10^−3^ m of thickness.

Figure 1 shows the kinetics of peach fruit drying in slices of 1, 2, and 3 × 10^−3^ m. Water content in samples with 1 × 10^−3^ m of thickness decreased faster compared with the other samples for 40 min and attained values of 0.05 kg of H_2_O/kg of dry solid. This process took 50 and 60 min for samples with 2 × 10^−3^ and 3 × 10^−3^ m, respectively. Similar results have been reported in mango slices of (*Mangifera indica* L.) 2 × 10^−3^ m after 1 h of drying (35). The evolution observed in the drying process for slices of 1 × 10^−3^ m thickness were higher than others works reported in the literature in the drying of breadfruit slices (*Artocarpus communis*) (21) and in mango slices (35), due to high sugar content in peach fruits and their interaction with infrared energy supplied by the RWD which creates a thin caramelized barrier blocking the water movement from the inner side of the samples to the air, increasing the drying time in thinner slices.

#### 3.2.2 RWD of peach pulp surpluses with maltodextrin

Table 4 shows CCD experimental design for RWD-MD where, for all experiments, moisture content was reduced until a range between 5% to 17%, and the water activity was reduced from 0.985 in the fresh peach until levels between 0.36 to 0.786, these results were highly influenced by MD added and its hygroscopic effect on peach pulp. Table 5 shows the ANOVA and their correspondent response surface analysis of RWD-MD, where time and MD have a strong effect on the moisture content and water activity (*p* < 0.05), these results showed that, at all MD levels, carrier addition improved the moisture content and water activity reduction.

**Table 5:** Experimental design CCD augmented for RWD of peach fruit pulp with the addition of maltodextrine (MD) and results for water content on dry basis (*H*, %) and water activity (*a*_w_).

Order	MD (X_1_)^*^	Time (X_2_)	MD (kg/kg)	Time (min)	*H* (%)^**^	*a*_w_
1	0	+*α*	0.23	180.7	5.63	0.413
2	0	−*α*	0.23	39.3	17.27	0.787
3	0	0	0.23	110	6.92	0.482
4	−1	+1	0.15	160	5.18	0.363
5	−1	−1	0.15	60	9.95	0.572
6	+1	+1	0.30	160	6.86	0.522
7	+1	−1	0.30	60	14.67	0.767
8	0	0	0.23	110	7.88	0.529
9	0	0	0.23	110	7.40	0.510
10	+*α*	0	0.33	110	9.31	0.672
11	−*α*	0	0.12	110	5.85	0.389

^*^Where *α* is the axial distance, $\alpha ={\left(2^{k}\right)}^{\frac{1}{4}}$ , *k* is the number of factors. ^**^Dry basis.

High regression coefficients (*R*^2^) were found for RWD-MD which indicates a good correlation as they explain more than 98% of the variability for moisture content (*R*^2^ = 0.9826) and (*R*^2^ = 0.9894). As seen in Table 5, in both regression models, time has the strongest effect decreasing moisture content and indicating that RWD has great advantage in time process reduction.

**Figure 2:**
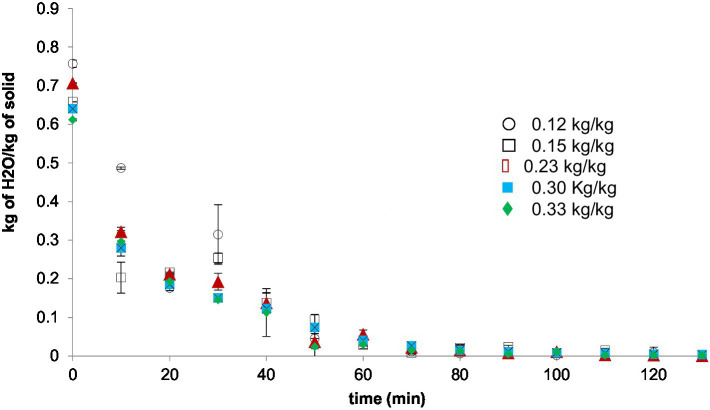
Kinetics of moisture content for RWD with maltodextrin addition (RWD-MD) at different levels (0.12–0.33 kg MD/kg peach pulp).

Figure 2 shows RWD kinetics for peach pulp mixed with MD where after 40 min all the samples attained a water reduction between 0.12 to 0.14 kg of H_2_O/kg of dry solid and reductions from 0.84 kg of H_2_O/kg solid from the fresh pulp. An ANOVA revealed that there are non-significant differences between the samples (*p* = 0.8965). These values were higher than the RW of peach slices for 1 to 2 × 10^−3^ m at the same time of processing and other studies (21,35), showing that MD has a significant effect on the drying process without dependence on the amount mixed with peach pulp. This effect could be due to the increase of carbohydrates concentration in samples which under heat conditions may cause caramelization on the surface and therefore cause a reduction of the water mass transfer from the inner to the surface and getting up the drying time as can be observed in Figure 2.

High regression coefficients (*R*^2^) were found for RWD-MD, which indicates a good correlation as they explain more than 98% of the variability for moisture content (*R*^2^ = 0.9826) and $a_{w}$ (*R*^2^ = 0.9894). As shown in Table 4, in both regression models, time has the strongest effect, decreasing moisture content and $a_{w}$ indicating that RWD has great advantage in time process reduction.

#### 3.2.3 Oven drying

The results for hot air or oven drying in terms of moisture content and water activity were 3.59 ± 0.74 (db) and 0.28 ± 0.02, respectively. These values were lower than those obtained using RW in this study and other agricultural products reported in the literature (64–66). However, the residence time was approximately 23 h, which makes it an inefficient process in terms of time and energy consumption.

#### 3.2.4 Diffusion coefficients

Table 6 shows the diffusion coefficients (*D*_eff_) for RWD in slices and RWD-MD, and the highest values were found for the RWD slices with 1 × 10^−3^ m (1.63 × 10^−6^ m^2^ s^−1^). This result showed that the *D*_eff_ data increased with the thickness. The possible cause of this outcome can be attributed to the phenomenon of shrinkage, as the shrinkage of food leads to a change in the distance required for the transportation of water molecules. Similar results were noted during the RWD of feijoa slices (*Acca sellowiana* Berg), where the thinner thickness exhibited a greater *D*_eff_ compared with the samples with a thicker thickness (67). This behavior has been reported previously for potato (68) and *Aloe vera* slices (20). For RWD-MD, the greater *D*_eff_ has been observed when the MD concentration is at 11% (8.73 × 10^−8^ m^2^ s^−1^). This trend can be attributed to the lower solid concentration and the higher abundance of water, resulting in increased transportation velocity and has been described before in the drying of *Passiflora edulis* puree mixed with carboxymethyl cellulose (69).

**Table 6:** Effective diffusion coefficients (*D*_eff_) for RWD slices and RWD with MD addition and a thickness of layer of 2 × 10^−3^ m.

RWD method		*D*_eff_ (m^2^ s^−1^)
Slices, thickness (m *×* 10^−3^)	1	2,50 × 10^−8a^
	2	7,82 × 10^−8a^
	3	1,33 × 10^−6b^
MD addition (kg/kg), thickness layer: 2 × 10^−3^ m	0.12	8,73 × 10^−8a^
	0.15	6,46 × 10^−8ac^
	0.23	7,42 × 10^−8ac^
	0.30	6,47 × 10^−8ac^
	0.33	7.66 × 10^−8ac^

Different lowercase letters (a, b, and c) represent significant differences at 0.05.

#### 3.2.5 Drying methods and their effect on the color attributes

Table 7 shows the effect on color attributes (L^*^, a^*^, b^*^, C^*^, and *h*) in dependence of the drying process applied. The effects of RWD-MD and RWD-slices on the L^*^ value were significantly different in opposite ways influenced by the addition of MD; in the case of RWD-MD, luminosity was reduced, and dark appearance on samples could be due to time exposition to infrared heat treatment and the presence of sugar compounds from the peach pulp surpluses followed by the MD solubilization and caramelization creating brown and dark aggregates on the surface. OD and RWD slices showed lower L^*^ values compared with fresh peach, showing the drying effect on the luminosity, and both revealed the same effect; nonetheless, RWD methods show better cost-efficient results in terms of processing time when compared with the conventional oven drying OD (time: ~24 h). In general, all the drying methods showed significant differences in L^*^ compared with unprocessed peach.

**Table 7:** Overall drying process and their effects on the moisture content, water activity, and color attributes.

Drying process	Physical		Color attributes					
	Moisture content (%)^*^	Water activity (*a*_w_)	L^*^	a^*^	b^*^	C^*^	*h*	Δ*E*
RWD slices	9.45 ± 0.05^a^	0.59 ± 0.01^a^	55.30 ± 2.03^a^	11.53 ± 1.85^a^	27.87 ± 2.82^a^	30.19 ± 3.31^a^	22.39 ± 1.22^a^	30.31 ± 3.40^a^
RWD-MD	5.91 ± 0.90^b^	0.42 ± 0.06^b^	32.51 ± 4.18^b^	2.29 ± 0.90^b^	9.48 ± 2.04^b^	9.77 ± 2.13^b^	13.41 ± 4.14^b^	48.48 ± 2.96^b^
Oven Drying	3.59 ± 0.74^c^	0.28 ± 0.02^c^	56.11 ± 1.73^a^	25.77 ± 3.46^c^	47.82 ± 1.61^c^	51.82 ± 0.63^c^	22.43 ± 4.17^a^	21.86 ± 2.51^a^
Fresh peach	89.22 ± 0.04^d^	0.99 ± 0.00^e^	74.22 ± 4.11^d^	19.09 ± 2.98^c^	58.78 ± 6.87^d^	61.81 ± 7.38^d^	17.95 ± 1.25^a^	—

Superscript letters different in the same column means significance difference with *p* < 0.05. Moisture content on dry basis.

The degree of redness and greenness (a^*^) values was highly reduced in RWD-MD samples and showed higher difference when compared with the peach control; OD showed values without differences against the control followed by RWD slices, where a^*^ values were close to the control under a less significant difference (LSD) test, and this behavior was observed in b^*^ values which is a good indicator to distinguish differences in yellowness of peach samples affected by the drying conditions.

All drying treatments, overall, exhibited distinctions from the control group and it shows a possible degradation or oxidation of carotenoids, which affected the natural yellow color of peach fruit and causes the arising of brown color as described in RWD production of mango powder (70) and hot air drying of sea buckthorn berries (*Hippophae rhamnoides* L.) (37).

The vividness of the color described by Chroma attribute (C^*^) had the highest difference between fresh peach and RWD-MD (LSD = 52.04) but had a small difference with OD (LSD = 9.99) followed by RWD slices treatments (LSD = 31.61), showing that the use of carriers decreases the vividness of peach during the drying process. The values for hue angle (*h*) showed no difference between fresh peach and RWD slices and OD (*p* > 0.05), and the lowest values in hue angles were obtained for RWD-MD due to loss in natural yellowness and redness induced by the combination of drying process and the addition of MD. An evaluation of how color changes affected the global perception was made using the total deviation (Δ*E*) (see Table 6), where all treatments showed big changes against the control (fresh peach), and the lower difference was observed with OD (21.86 ± 2.51) followed by RWD slices (30.31 ± 3.40), higher Δ*E* results were observed for RWD-MD, showing values of 48.48 ± 2.96 due to the addition of MD, and similar results were reported for RWD of banana slices (1) and buckthorn berries (37). All the treatments affected the color global perception, and a not trained customer could detect these changes easily, applying the rule of acceptance (Δ*E* ≤ 2.56) (51); nonetheless, in this study, the most suitable products were obtained by OD and RWD slices.

**Figure 3:**
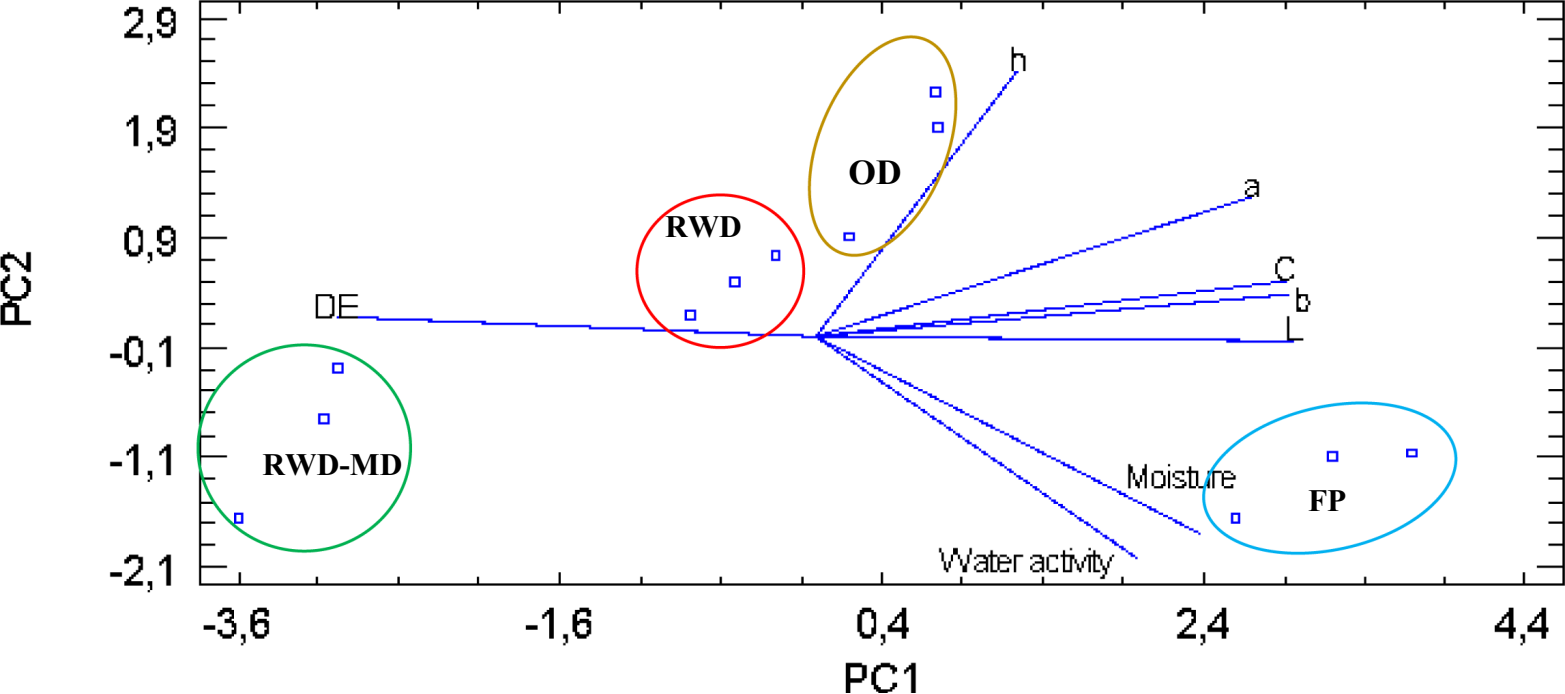
Biplot of principal component analysis (PC1) versus (PC2) of the effect of drying process on the physical and color attributes of peach. Where: OD, oven drying; RWD-MD, Refractance Window^™^ drying with maltodextrine; RWD-slices, Refractance Window^™^ drying in slices; FP, fresh peach.

A multivariate analysis by principal component analysis (PCA) was made to identify the total variance implied at all the response variables using the weighting method of standardization. Seven principal components (PCs) were detected, accounting for 100% of the total variability. The first two of them accounted for 93.80% of the variance in the seven variables (PC1 = 72.70; PC2 = 21.10) and the *biplot* (see Figure 3), showing the behavior for PC of physical properties and color attributes.

Data observation suggests that, generally, L^*^, a^*^, b^*^, and Chroma (C^*^) have a strong weight on positive quadrant for PC1, showing a good contribution on the total variance and being the most important attributes for the analysis; hue angle has a strong weight on PC2 on the positive quadrant, showing a close effect with the color attributes, and on the other hand, the moisture content and water activity showed strong weight on PC1 and PC2 in the negative quadrant, suggesting that when moisture content and water activity increase luminosity, yellowness and the hue angle decrease. RWD-MD is placed in the opposite side of FP, while data on RWD slices are quite close to the center and closer to OD, indicating that under multivariate analysis, the most suitable drying process contributing to less variance was OD and RWD slices. RWD-MD has the opposite effect over the attributes L^*^, a^*^, b^*^, C^*^, and *h* due to MD addition effect, which means MD has a strong negative effect on peach color dried product. DE (Δ*E*) showed an opposite and strong effect with the fresh pulp and the color coordinates L^*^, b^*^, C^*^, and a^*^; nonetheless, it was closer to the RWD-MD data group, explaining the effect of MD addition and the total color differences results.

#### 3.2.6 Vitamin C and carotenoids

Vitamin C and carotenoids were measured only in the RWD-slices based on the color and PCA results. Vitamin C was not detected instrumentally in any of the treatments for the different sliced peach samples, and this may be caused by the thermo-sensitivity of vitamin C, which degrades at temperatures over 60°C and a relative exposure to light. During RWD slices, the average water temperature reached 86°C, which emits sufficient heat rate that would cause thermal degradation of vitamin C. These results are in agreement with other authors who have carried out RW of materials such as pomegranate leather, jackfruit pulp, and sea buckthorn, where the authors found a loss of vitamin C between 30 and 74% compared with the fresh product (29, 37, 65).

The β-carotene contents for RWD slice results were 175.88 ± 2.11, 135.03 ± 3.73, and 104.83 ± 0.86 for peach slices of 1, 2, and 3 × 10^−3^ m, respectively, with carotene loss compared with fresh peach of 35, 50, and 62%, respectively. The higher amount of β-carotene was found in the thinner samples, showing that at high temperature (86°C), increased thickness had a negative impact on the carotenoid content based on heat, light, and oxygen exposition during the RW process, and these results had a similar trend compared with other studies published on RW of jackfruit and passion fruit (29, 69). A recommendation to improve the vitamin C and carotenoid content can be a pretreatment of the peach samples with an ascorbic acid solution before the RWD (64).

#### 3.2.7 Sensory analysis

In this study, we employed principal components analysis (PCA) to investigate the impact of two different drying methods, RWD in slices and OD, on the overall perception and color properties of peach slices in comparison to fresh peaches. Our findings indicate that RWD and OD treatments exhibited reduced variance contributions in terms of overall perception and Δ*E* color results when compared with fresh peaches.

To further evaluate the effect of storage time on sensory acceptability, we conducted a sensory test on samples subjected to room temperature storage (25°C) for a period ranging from 1 to 45 days.

**Figure 4:**
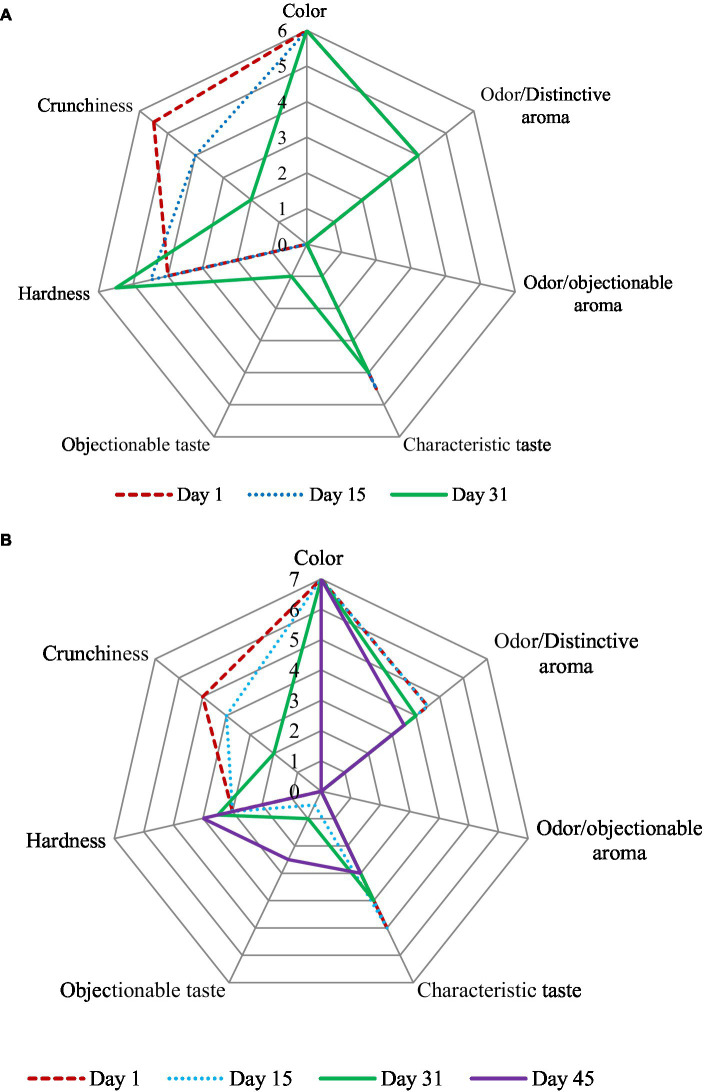
Sensory analysis acceptability scores in dependence of time (1, 15, 31, and 45 days of storage at 25°C) for a = oven drying and b = RWD of peach.

For samples subjected to OD (see Figure 4A), our results suggest that sensory quality remained acceptable for up to 15 days of storage. Beyond this duration, a decline in sensory attributes, characterized by objectionable taste, hardness, and crunchiness, became evident, ultimately resulting in an overall sensory decline and rejection after 30 days of storage.

In contrast, samples subjected to RWD and stored under the same conditions (see Figure 4B) exhibited acceptable sensory quality for an extended period of up to 30 days of storage. However, beyond this timeframe, sensory attributes deteriorated, with the emergence of objectionable taste, hardness, and a loss of crunchiness. Notably, our sensory panel did not reject any of the products, and products subjected to RWD exhibited high crunchiness, appealing appearance, color, odor, and taste characteristics. These findings suggest that RWD products hold significant potential for entry into the snack market, owing to their favorable sensory attributes.

In summary, our study underscores the differences in sensory quality and shelf life between peach slices subjected to RWD and OD treatments. The sensory attributes observed in RWD products position them as promising candidates for the snack market, offering a longer shelf life and desirable sensory characteristics.

## 4 Conclusion

In conclusion, the study demonstrates the effectiveness of RWD in slices as it reduces water activity by nearly 40% for peach slices. The drying times for RWD, ranging from 40 to 60 min, show moisture reduction values of approximately 0.05 kg of H_2_O/kg of dry solid. The incorporation of MD carrier in RWD reduces the moisture content to approximately 0.13 kg of H_2_O/kg of dry solids in just 40 min. The high correlation for both RWD slices (0.705–0.865) and RWD-MD (0.983–0.989) in terms of moisture content and water activity demonstrates strong agreement between experimental data and regression models. Principal component analysis (PCA) identified key components associated with color attributes, water content, and water activity.

Despite minimal impact on color attributes, RWD slices achieved substantial reductions in moisture content and water activity compared with fresh peach samples, even surpassing oven drying. However, there was thermal degradation of vitamin C, rendering it undetectable in dried peach slices. Additionally, a reduction in β-carotene content up to 62% emphasizes on the recommendation of pretreatment with ascorbic acid before drying.

The sensory acceptability assessment indicated that RWD products maintained good acceptability even after 31 days of storage at 25°C. These conclusive findings highlight the potential of RWD on the consumer’s acceptability of dried peach products, offering promising opportunities for surplus peach utilization in the food market.

## Data availability statement

The original contributions presented in the study are included in the article/Supplementary material, further inquiries can be directed to the corresponding author.

## Ethics statement

As this study did not require the use of humans, animals, or others, this manuscript does not require an ethics statement.

## Author contributions

EL-A: Data curation, Formal analysis, Investigation, Resources, Writing – review & editing. FR-R: Formal analysis, Methodology, Writing – original draft. JP-F: Formal analysis, Investigation, Methodology, Resources, Writing – review & editing. AL-P: Conceptualization, Methodology, Resources, Supervision, Validation, Writing – review & editing.
