# Supplementary material for: Effect of Refractance Window™ and oven drying on physicochemical and sensory properties of peach (Prunus persica L.) surplus
Source: Front Nutr. 2024 Apr 23;11:1307423. doi: 10.3389/fnut.2024.1307423 (PMC11075169; doi:10.3389/fnut.2024.1307423)
Supplement: Supplementary file 1 [file Data_Sheet_1.pdf]

Effect of Refractance Window^{TM} Drying and Oven Drying on Physicochemical and Sensory properties of Peach (*Prunus persica* L.) surplus

SUPPLEMENTARY MATERIAL

VITAMIN C. CHROMATOGRAMS

CONTROL NUMBER: C230907-066

PRODUCT: PEACH – RWD

SAMPLE ID: PEACH (*Prunus persica* L.)

CHROMATOGRAPHIC ANALYSIS: Vitamin C determination by HPLC

SAMPLE MASS: 25 g

DESCRIPTION: Dried peach with 3 different thickness (1, 2 y 3 mm).

MOBILE PHASE: Buffer KH_2PO_4 100 mM, pH= 2.5.

FLOW RATE: 1 mL/min

COLUMN: #1 – XDB C18

TEMPERATURE: 25 °C

DETECTOR: #1 DAD

LENGTH: 245 nm – Reference: 4 nm.

STANDARD: L-(+)-Ascorbic acid (Sigma Aldrich, 47863)

**Sample**	**Result**	**Unit**	**Analysis**
Peach – 1mm	N.D	mg/100 g	Vitamin C
Peach – 2 mm	N.D	mg/100 g	Vitamin C
Peach – 3 mm	N.D	mg/100 g	Vitamin C

N.D. Not detected (Vitamin C is outside the detection limits depending on the method and equipment conditions).

Effect of Refractance Window^{TM} Drying and Oven Drying on Physicochemical and Sensory properties of Peach (*Prunus persica* L.) surplus

SUPPLEMENTARY MATERIAL

VITAMIN C. CHROMATOGRAMS

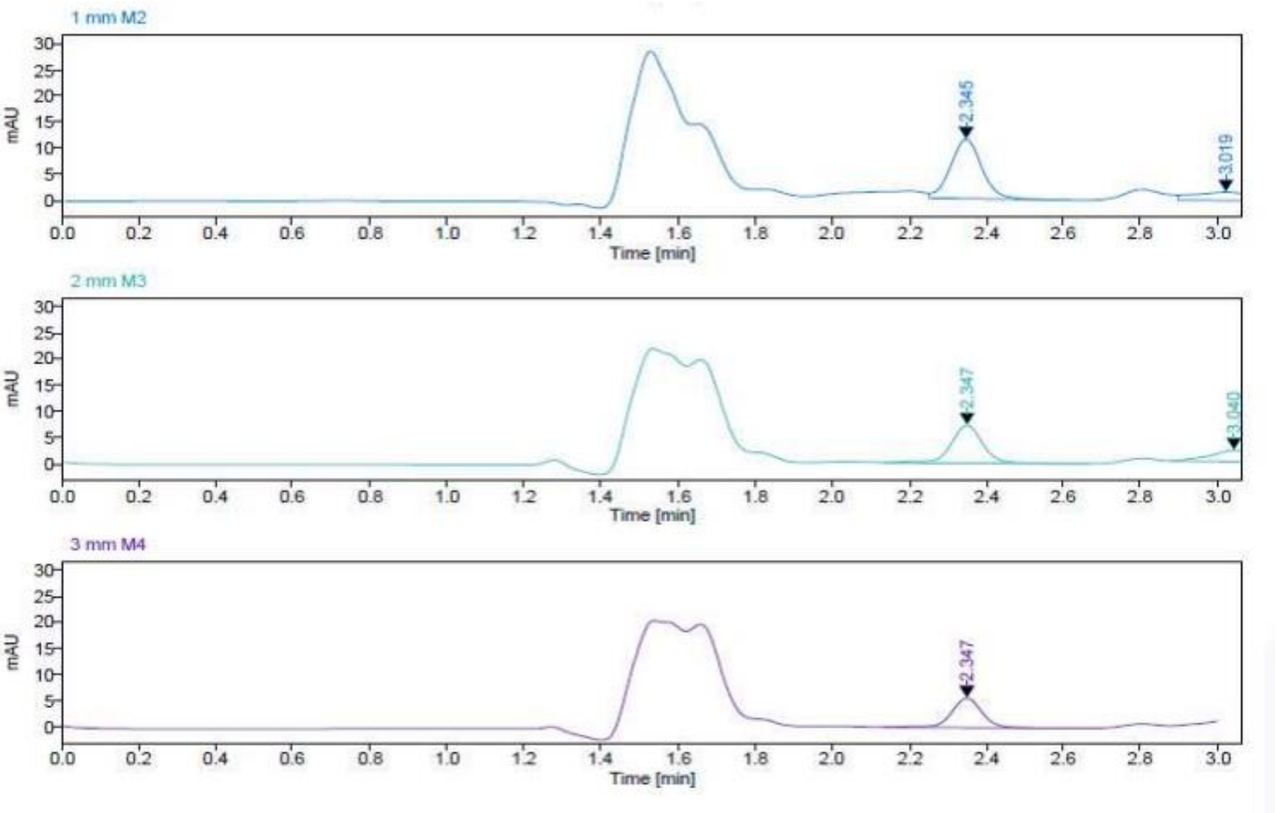

Effect of Refractance Window^{TM} Drying and Oven Drying on Physicochemical and Sensory properties of Peach (*Prunus persica* L.) surplus

SUPPLEMENTARY MATERIAL

QUALITY CRITERIA CLASSIFICATION FOR FRUITS AND VEGETABLES IN COLOMBIA

According to the Colombian Technical Standard NTC 1291 of 1975, a series of quality requirements have been defined that fruits and vegetables must meet in order to be marketed fresh. This norm has created three types of quality terms: Extra quality, first quality and second quality. Those that do not meet the second quality criterion are considered as surplus and are marketed in low value markets or according to the interests of the interested parties (see Table 1 and 2).

Table 1. Quality criteria classification according with NTC 1291:1977 (1)

Quality criteria	Extra, first and second quality
Minimum size	In mm
Shape	In %
Consistence	In %
Color	In %
Serious damage	In %
Signs of rotting	In %
Minor damages	In %
- Bruises	In %
- Spots	In %
- Superficial or healed wounds	In %
- Pest damage	In %
- Cumulative tolerance	In %

Table 2. Quality parameters by commercial category for Colombian peaches (2)

Quality category	Equatorial diameter (mm)	Fresh weight (g)	°Brix
Extra	> 70	186 ± 13	13.0 ± 2.5
First (I)	60 – 70	145 ± 17	11.0 ± 1.6
Second (II)	50 – 70	90 ± 12	11.1 ± 1.9
Surplus	< 50	69 ± 12	-

References:

1. Instituto Colombiano de Normas Técnicas – ICONTEC. NTC 1291. Fresh fruits and vegetables. Generalities. (1977)5. https://vdocuments.mx/ntc-1291-fruta-y-hortalizas-generalidades.html

2. Bastidas Parrado AL, Puentes Montañez GA, Lemus Cerón A. Parámetros de calidad durante la cosecha de durazno (Prunus persica L . Batsch cv . "Rubidoux ”). *Rev Investig Agrar y Ambient* (2015) 6:145–154.
